# Severe COVID-19 Myocarditis in a Young Unvaccinated Patient

**DOI:** 10.7759/cureus.37942

**Published:** 2023-04-21

**Authors:** Sindhu Chandra Pokhriyal, Muhammad Nabeel Pasha, Pooja Devi, Hadeeqa I Bhatti, Ruchi Yadav

**Affiliations:** 1 Internal Medicine, One Brooklyn Health, New York, USA; 2 Pulmonology and Critical Care, One Brooklyn Health, New York, USA; 3 Internal Medicine, Shifa International Hospital, Islamabad, PAK; 4 Hematology and Oncology, One Brooklyn Health, New York, USA

**Keywords:** device, impella, ecmo, mechanical, complication, covid, myocarditis

## Abstract

Coronavirus disease 2019 (COVID-19) myocarditis is a rare but serious complication of severe acute respiratory syndrome coronavirus 2 (SARS-CoV-2) infection and has been associated with high-case fatality. For a very long time, since the beginning of the pandemic, there were no definitive guidelines to diagnose and manage this condition, probably secondary to the gaps in understanding the exact pathophysiology of the disease. We present the case of a young, unvaccinated female, with no comorbidities, who had an aggressively progressive COVID-19 myocarditis that was fatal. The patient presented with exertional dyspnea of two days duration and was found to be tachycardic with a heart rate ranging between 130-150 beats per minute. A nasopharyngeal swab for SARS CoV-2 was positive and a bedside echocardiogram showed a low ejection fraction of 20%. Within hours of presenting, she experienced a rapid decompensation requiring intubation. Due to fulminant myocarditis with cardiogenic shock, the patient was planned for cardiac catheterization, Impella placement, and extracorporeal membrane oxygenation (ECMO) support. The cardiac catheterization revealed non-obstructive coronary arteries and the hemodynamics suggested biventricular failure. However, around the time of the cardiac catheterization procedure, she had two events of cardiac arrest with pulseless electrical activity and unfortunately could not be revived after the second arrest despite all resuscitative efforts.

## Introduction

Coronavirus disease 2019 (COVID-19) is a respiratory infection that first broke out in Wuhan, China in December 2019 and rapidly spread to the entire world over the next few years [1]. Many countries have experienced a multi-wave pattern in the reported cases of COVID-19 over the last three years. The severity and complications of COVID-19 infections have varied across the different waves in different parts of the world [1,2]. COVID-19 infection is known to have a variable clinical presentation, ranging from asymptomatic disease to devastating multiorgan failure and death. Complications of COVID-19 are many and include acute respiratory distress syndrome (ARDS), hypercoagulability, endothelial injury, vasculitis, cytokine storm, pulmonary embolism, stroke, and myocarditis [2].

The cardiac complications of COVID-19 include acute coronary syndrome, atrial and ventricular arrhythmias, Takotsubo cardiomyopathy, myocarditis, and cardiogenic shock. Myocarditis is the focal or diffuse inflammation of the heart muscle, which can lead to disastrous complications like heart failure and death. Viral infections such as enteroviruses, parvovirus, and herpes are known as the commonest cause and triggers of myocarditis. Albeit rare, myocarditis is now a well-recognized and serious complication of COVID-19 [3]. Centers for Disease Control and Prevention (CDC), in their data from over 900 different hospitals between March 2020 and January 2021, found a nearly 16-fold increase in the risk of myocarditis in patients with SARS-CoV-19 when compared to the average population [4]. Lala et al. in their study found that some degree of myocardial injury can be present in up to 30% of hospitalized patients with moderate to severe COVID-19 [5].

In May 2022, the American College of Cardiology outlined an expert consensus pathway streamlining the diagnosis and management of COVID-19 myocarditis [6].

## Case presentation

A 31-year-old female with no past medical history presented to the emergency department (ED) in the month of January 2023 with chest pain and generalized body pains for two days. The chest pain was described as substernal, 10/10 at its peak, radiating to the back, and made worse with breathing. The patient also reported worsening exertional dyspnea and palpitations and that the symptoms had worsened over two days prompting the presentation to the ED. The patient was single, had no previous pregnancies, denied sick contacts, recent travel, oral contraceptive use, fever, hearing loss, tinnitus, visual disturbances, change in taste or smell, cough, sputum production, leg swelling, abdominal pain, blood in stool, constipation, diarrhea, nausea, vomiting, dysuria, increased frequency of micturition, neck pain, headaches or easy bruising. She reported no history of smoking, alcohol use, or any other substance abuse. She was not on any medications and worked as a part-time online influencer. She had received all childhood vaccinations; the last vaccine she received was the meningococcal vaccine at the age of 15 years. However, she was not vaccinated against SARS-CoV-2 and had never received any annual seasonal influenza vaccines due to personal beliefs.

**Figure 1:**
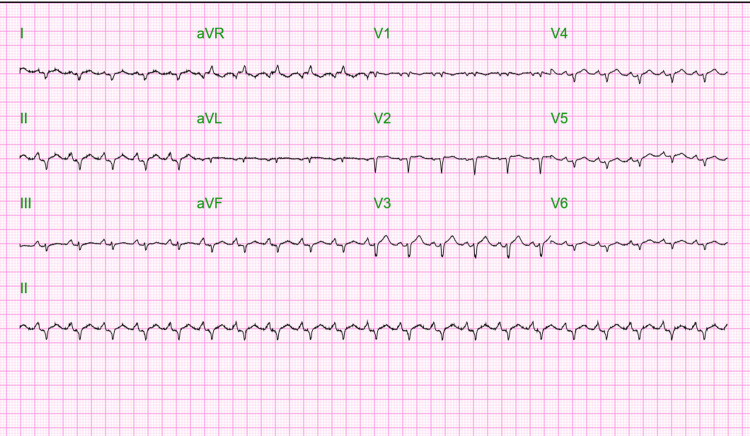
ECG of the patient taken at admission showed sinus tachycardia with a heart rate of 150 beats per minute.

The physical examination was significant for tachycardia ranging between 130-150/min. Her blood pressure (BP) was 112/82 mmHg, she was afebrile, not tachypneic, and she was saturating 98% on ambient air at presentation. Her BMI was 20.9 and the rest of the physical examination was unremarkable. Nasopharyngeal swab polymerase chain reaction (PCR) testing upon hospital admission was positive for SARS-CoV-2. Electrocardiogram (ECG) showed sinus tachycardia and right axis deviation (Figure 1).

**Figure 2:**
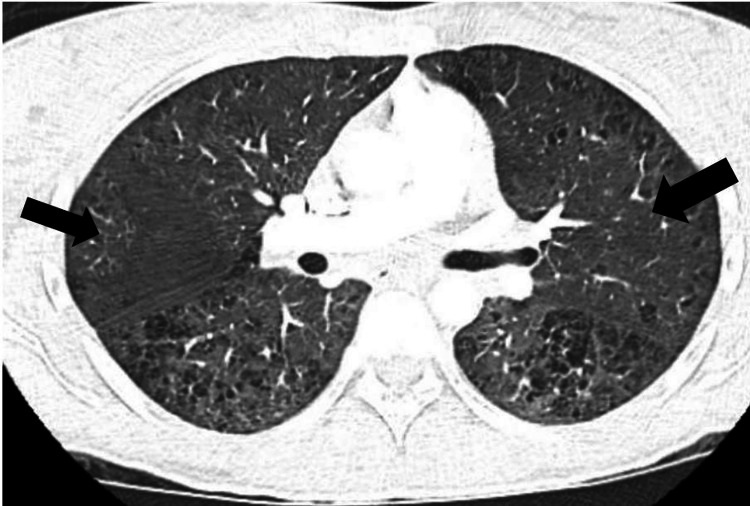
Typical appearance of cardiogenic pulmonary edema in bilateral lungs on CT chest

Initial troponin I was 1.3 ng/mL (reference range: <=0.034 ng/mL) and N-terminal pro brain natriuretic peptide (pro-BNP) was 6000 pg/mL (11.1 - 125.0 pg/mL). A bedside echocardiogram showed small pericardial effusion and a severely depressed ejection fraction of less than 20%, raising concerns for myocarditis. CT angiogram of the chest was negative for pulmonary embolism and showed typical findings of diffuse cardiogenic pulmonary edema (Figure 2).

The patient was initiated on intravenous dexamethasone and remdesvir for COVID-19 pneumonia and was admitted to the Critical Care Unit for close monitoring. She had a heart rate in the range of 130-150 beats per minute, with lactate and troponin trending up over the next few hours. Troponin increased to 6.520 ng/ml and lactate increased from 7.2 mmol/L to 9.9 mmol/L (reference range: 0.70-2.10 mmol/L). Within two hours of the presentation, she progressed to severe hypotension with a mean arterial pressure (MAP) ranging from 40-50 mmHg, thereby needing support with four pressors (including noradrenaline, vasopressin, phenylephrine, and epinephrine). The arterial blood gas (ABG) done showed a PH of 7.06, PCO2 of 59 mmHg (normal range 35-45 mmHg), PO2 70 mmHg (normal range 80-110 mmHg), bicarbonate 16 (normal range 22-26 mmHg), and oxygen saturation of 83% (normal range 95-100%). The lactate further increased to >24 mmol/L. The patient was initially commenced on oxygen by high-flow nasal cannula at 60%. Soon after, due to worsening mental status, she was intubated for worsening hypoxic respiratory failure with encephalopathy.

Due to the provisional diagnosis of fulminant myocarditis with cardiogenic shock, the patient was taken for cardiac catheterization and possible placement of mechanical heart support such as Impella or extracorporeal membrane oxygenation (ECMO) support. Before the procedure, just seven hours after admission, the patient had the first cardiac arrest with the primary rhythm of pulseless electrical activity (PEA). Advanced cardiac life support (ACLS) protocol was initiated and return of spontaneous circulation (ROSC) was achieved. Post ROSC she underwent a left ventriculogram (LVG), coronary angiogram, and right heart catheterization (RHC). LVG showed global hypokinesis with severely reduced ejection fraction of around 10%. The coronary arteries were noted to be patent. RHC findings were consistent with biventricular failure, with pulmonary capillary wedge pressure (PCWP) of 25 mmHg (normal range: 6-12 mmHg), Cardiac index or CI of 0.9 L/min/mm^3^ (normal >2.4L/min/mm^3^) and right atrial pressures of 23 mmHg (normal range: 2-6 mmHg). Femoral artery anatomy was found to be unsuitable for an Impella device insertion, and hence cardiothoracic surgery was contacted for placement on ECMO support. However, unfortunately, at this time, the patient had another cardiac arrest, again with a rhythm of pulseless electrical activity. At this time the patient could not be revived despite all resuscitative efforts. 

## Discussion

Cardiovascular involvement in COVID-19 infection has been associated with higher morbidity and mortality. COVID-19 myocarditis can have a fulminant and devastating course and is said to be responsible for nearly 7% of all COVID-19-related deaths [6]. Although COVID-19 myocarditis is generally thought to be rare, the precise estimation of its incidence has been limited by variation in reporting and diagnostic criteria, as well as by the infrequent application of the gold-standard diagnostic endomyocardial biopsy. The incidence is estimated to be as high as 30% of all moderate to severe COVID-19 hospitalizations [5]. According to one review by Haussner et al., where they reviewed 51 cases of COVID-19 myocarditis, the mortality associated with COVID-19 myocarditis was as high as 14%. They also reported that more than half (58%) of people with COVID-19-associated myocarditis also suffered from other comorbidities such as high blood pressure, diabetes, obesity, asthma, or chronic obstructive pulmonary disease (COPD) [7,8]. 

The mechanism of COVID-19 myocarditis is still not well understood [9,10]. Both direct myocyte injury during the cytokine storm as well as the binding of angiotensin-converting enzyme 2 (ACE2) spike protein on cardiomyocytes to induce myocyte injury, have been proposed as possible mechanisms of COVID-19 myocarditis [9,10].

Myocarditis is known to be a complication of COVID-19 vaccination, and in the initial phase of vaccination was one of the major feared complications of COVID-19 vaccination. Multiple studies, such as the one by Price et al., have concluded that the benefits of COVID-19 vaccination far outweigh the risk and drastically reduce the mortality from fulminant COVID-19 myocarditis [11]. In our patient, personal and community beliefs against COVID-19 vaccination were thought to have played a key role in the patient and her family refusing COVID-19 vaccinations during as well as after the pandemic. Awareness campaigns highlighting the benefits of COVID-19 vaccination are proposed to increase the uptake of COVID-19 vaccination in the community.

Clinical features of COVID-19 myocarditis include fever, chest pain, shortness of breath, palpitations, syncope, and arrhythmias. The symptoms of COVID-19 myocarditis are known to overlap with those of COVID-19-induced ARDS [9]. COVID-19 myocarditis is differentiated from ARDS primarily by characteristic features of acute heart failure, cardiogenic shock, or clinically significant ventricular arrhythmias [7,9]. In our patient, the characteristic presenting feature was that of sinus tachycardia, with a persistent heart rate ranging from 130-150 beats per minute despite initial fluid management. Although our patient also had a mild dry cough, her primary complaint was progressive dyspnea. Thus, it is important to note that in patients with COVID-19 infection, cardiopulmonary symptoms warrant and necessitate the workup for COVID-19 myocarditis as well [6].

Myocardial damage is known to present with ECG changes as well as elevated troponin levels [12]. Cardiac troponins like troponin I must be trended in patients with suspected COVID-19 infections. Initial workup in COVID-19 patients with cardiopulmonary symptoms entails performing the triad testing with ECG, cardiac troponin, and echocardiogram [6]. However, it is also important to keep in mind that elevated troponin may not always be present in COVID-19 myocarditis and the presence of normal troponin levels does not exclude the diagnosis of COVID-19 myocarditis [12]. ECG findings vary from nonspecific ST and T wave changes to diffuse ST elevations or ventricular arrhythmias [9]. If the triad testing is positive and the echocardiogram suggests acute onset reduced ejection fraction, cardiac catheterization must be considered, to rule out obstructive cardiac pathology. Cardiac magnetic resonance imaging (CMR) and myocardial biopsy, are resource-limited investigations but must also be performed wherever possible [6,7]. Echocardiogram shows features varying from left ventricular systolic impairment, right ventricular dysfunction, diastolic dysfunction, pulmonary hypertension, and biventricular failure. On CMR, myocardial enhancement is known to be the classical finding in COVID-19 myocarditis. This is secondary to underlying acute inflammation of the cardiac muscle as well as tissue hyperemia [13]. The gold standard criteria for the diagnosis of myocarditis is an endomyocardial biopsy. Myocyte necrosis or damage in the presence of inflammatory infiltrates is a hallmark of myocarditis, as defined by the Dallas criteria [14]. However, owing to being an invasive procedure, an endomyocardial biopsy is commonly not performed in most clinical scenarios. The latest consensus guidelines by the American College of Cardiology have suggested that endomyocardial biopsy is not mandatory for the diagnosis of COVID-19 myocarditis [6]. In our patient, triad testing done was positive and cardiac catheterization was performed which showed patent coronary arteries as well as features of biventricular failure in right heart catheterization. So although an endomyocardial biopsy could not be performed, all features strongly supported the diagnosis of COVID-19 myocarditis.

Immunosuppressants such as corticosteroids remain the first line in the management of COVID-19 myocarditis [6,9]. While mild to moderate cases of COVID-19 myocarditis are managed using remdesvir and dexamethasone, more severe COVID-19 myocarditis cases have been reported to be managed using intravenous immunoglobulin (IVIG) and intravenous methylprednisolone with a good response to this medical therapy [15-17]. As per a 2019 meta-analysis by Huang et al. comparing IVIG to corticosteroids for acute myocarditis, IVIG therapy improved mortality and recovery of left ventricular function [17]. However, more randomized controlled trials are required to strongly establish IVIG in the treatment algorithm of COVID-19 myocarditis. Our patient was managed with high doses of methylprednisolone.

**Figure 3:**
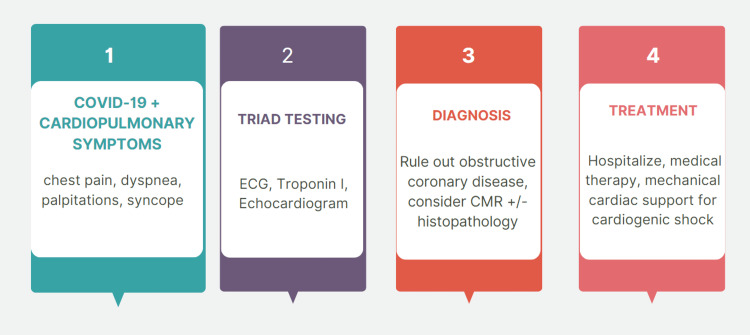
Diagnosis and management of COVID-19 myocarditis in adults. COVID-19: coronavirus disease 2019

Initial management of patients with COVID-19 myocarditis in cardiogenic shock involves using inotropes and mechanical ventilation [10]. Mechanical circulatory support (MCS) like ECMO, ventricular assist device, and intra-aortic balloon pumps (Impella) can be used to keep the blood pumping and support circulation during the phase of acute cardiogenic shock [18,19]. The Impella is one such device that has been used successfully in the treatment of severe myocarditis and cardiogenic shock in the past, both in conjunction with ECMO as well as independently [18-20]. Asakura et al. reported successfully using biventricular Impella to provide temporary mechanical circulatory support in a case of severe COVID-19 myocarditis in a young patient [20]. However, in our patient unfortunately the Impella could not be placed and although the plan was to go for ECMO support, this did not materialize due to the rapid deterioration and cardiac arrest from which the patient could not be revived. Once only utilized for patients with respiratory failure, ECMO is now being used increasingly on patients with cardiovascular compromise as well and must always be arranged swiftly, especially in fulminant cases [18.19]. A pictorial summary of the workup, diagnosis, and management of COVID-19 myocarditis is outlined in Figure 3 [6].

## Conclusions

COVID-19 myocarditis can have a fulminant and cataclysmic course and availability of a multidisciplinary team approach including prompt mechanical support to treat this fulminating condition is key in managing this potentially life-threatening disease. With the recent advent and presentation of more definitive and clear guidelines on COVID-19 myocarditis, hopefully, the mortality and morbidity from the illness is likely to see a favourable trend.
